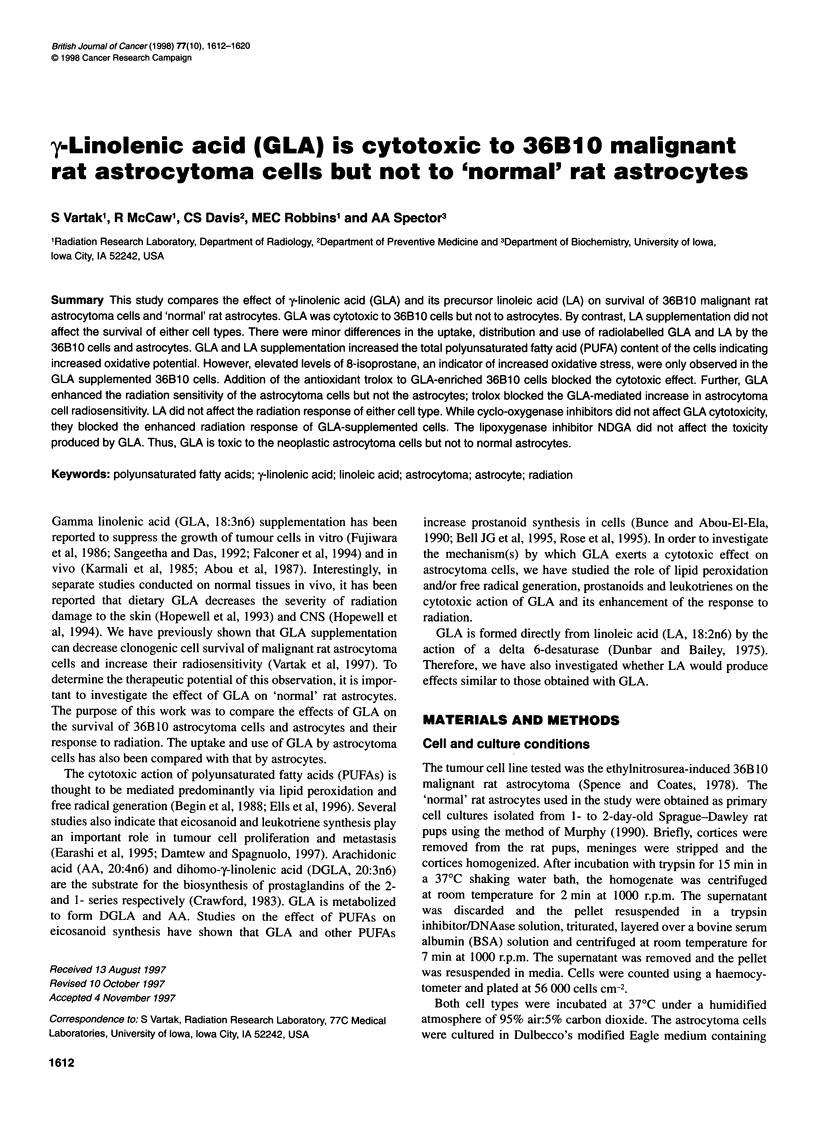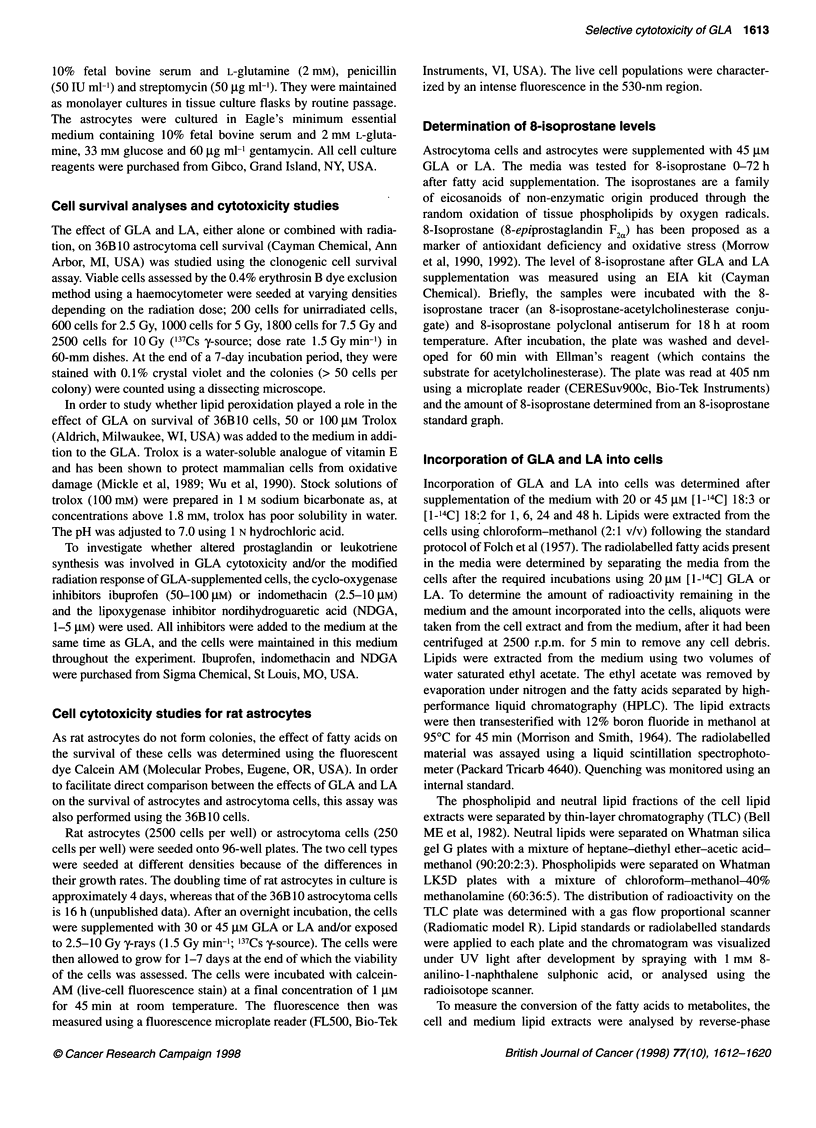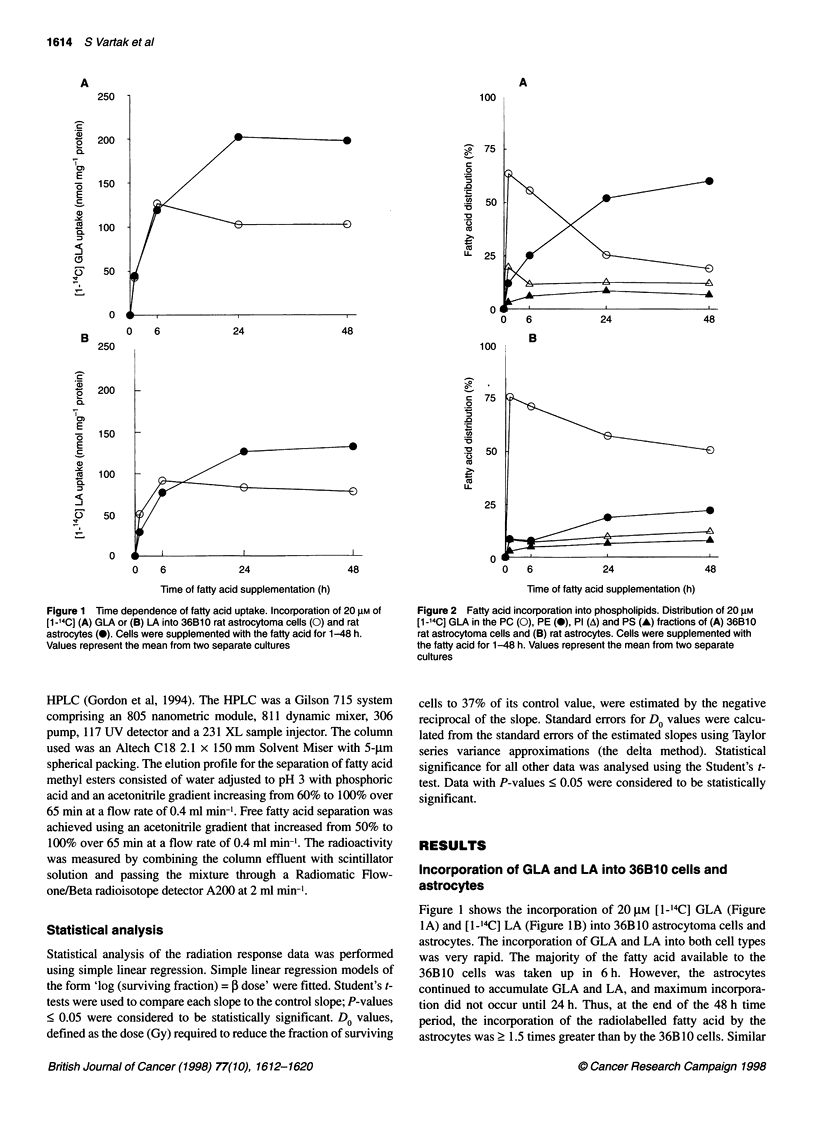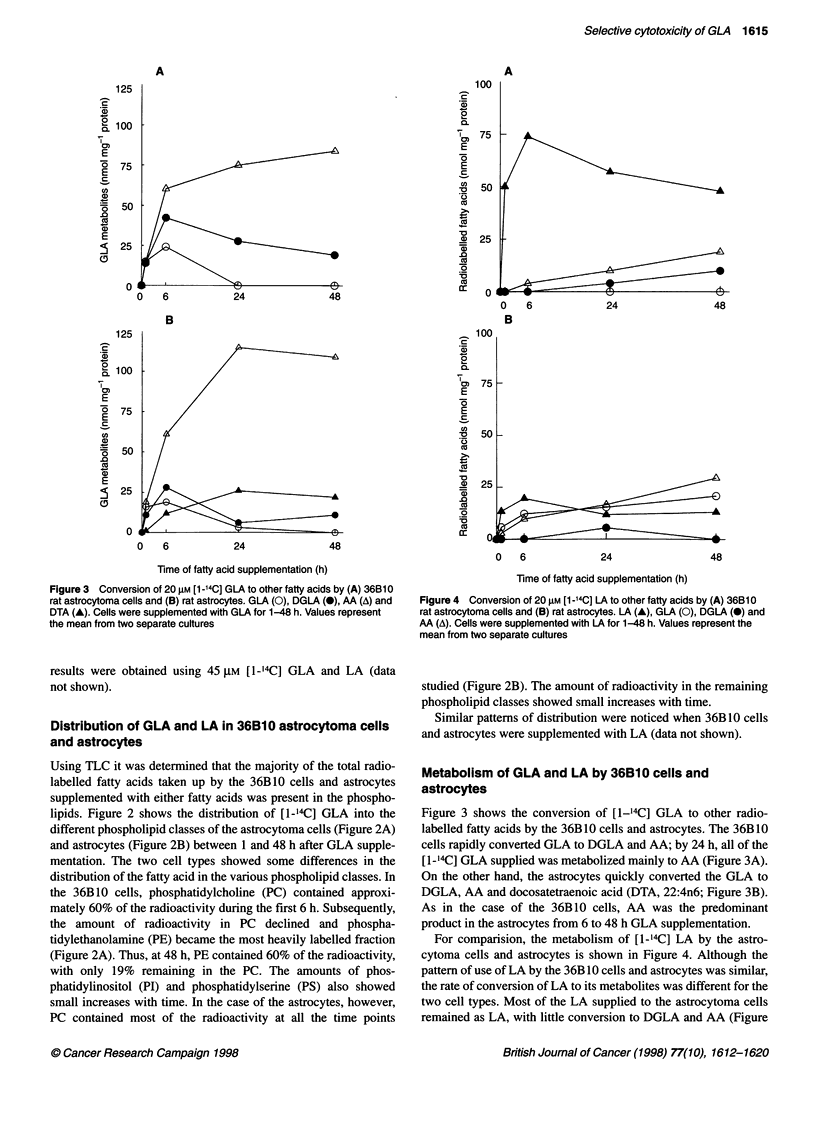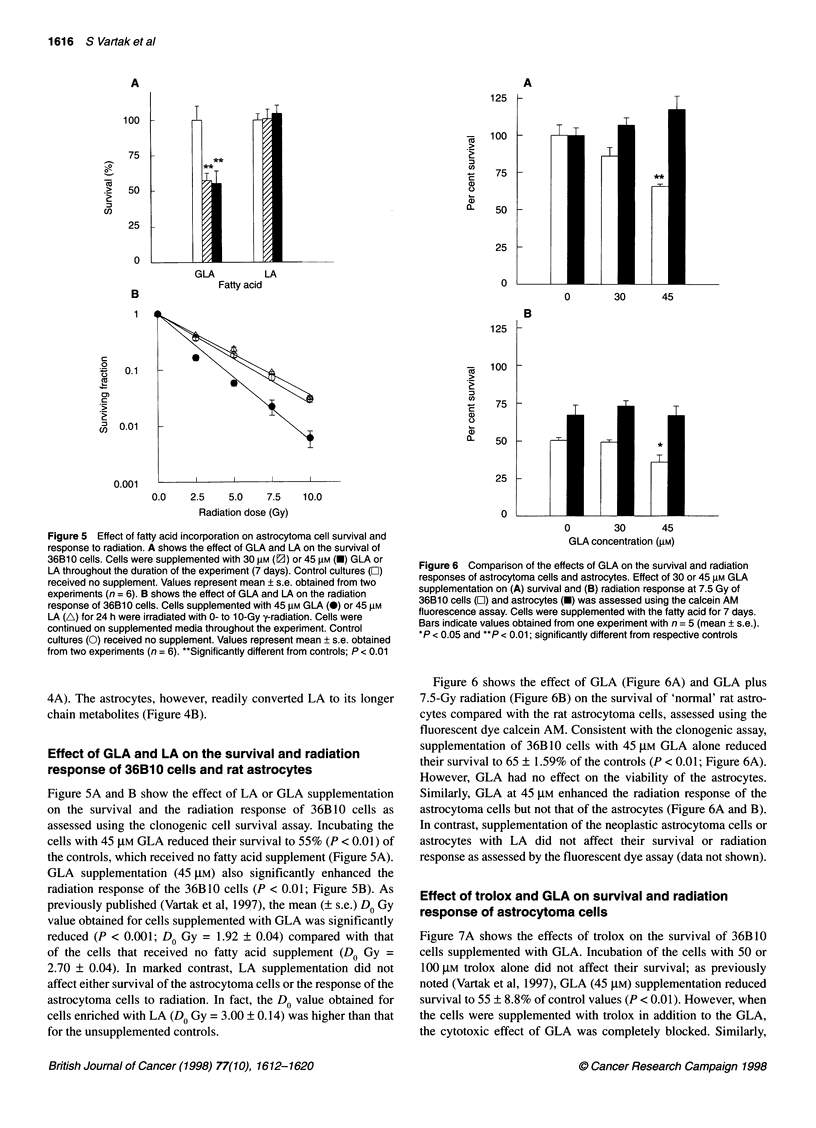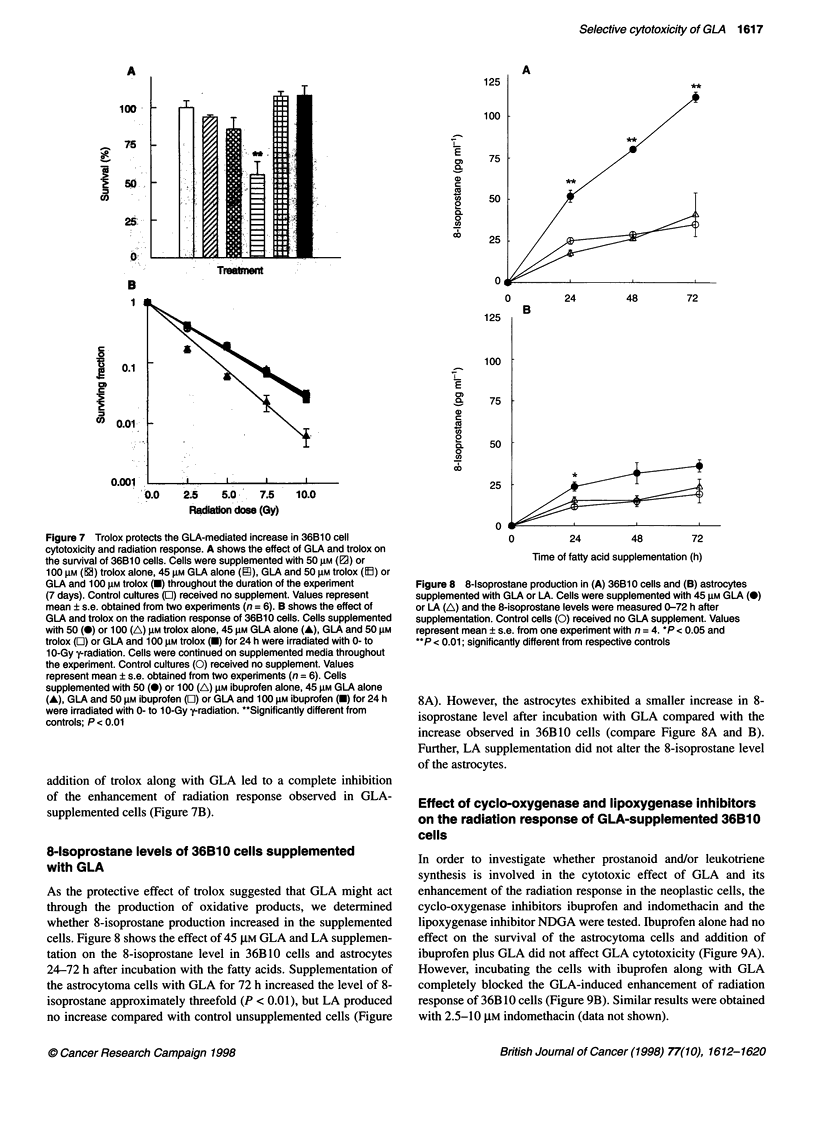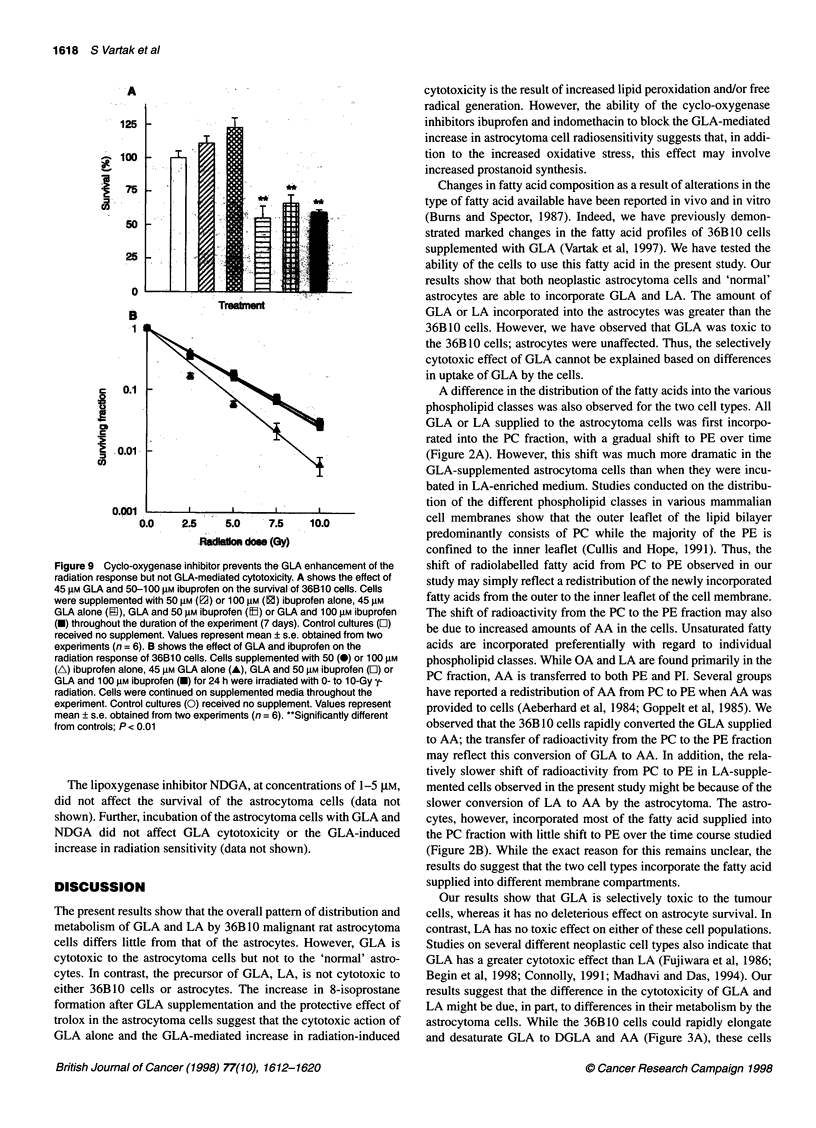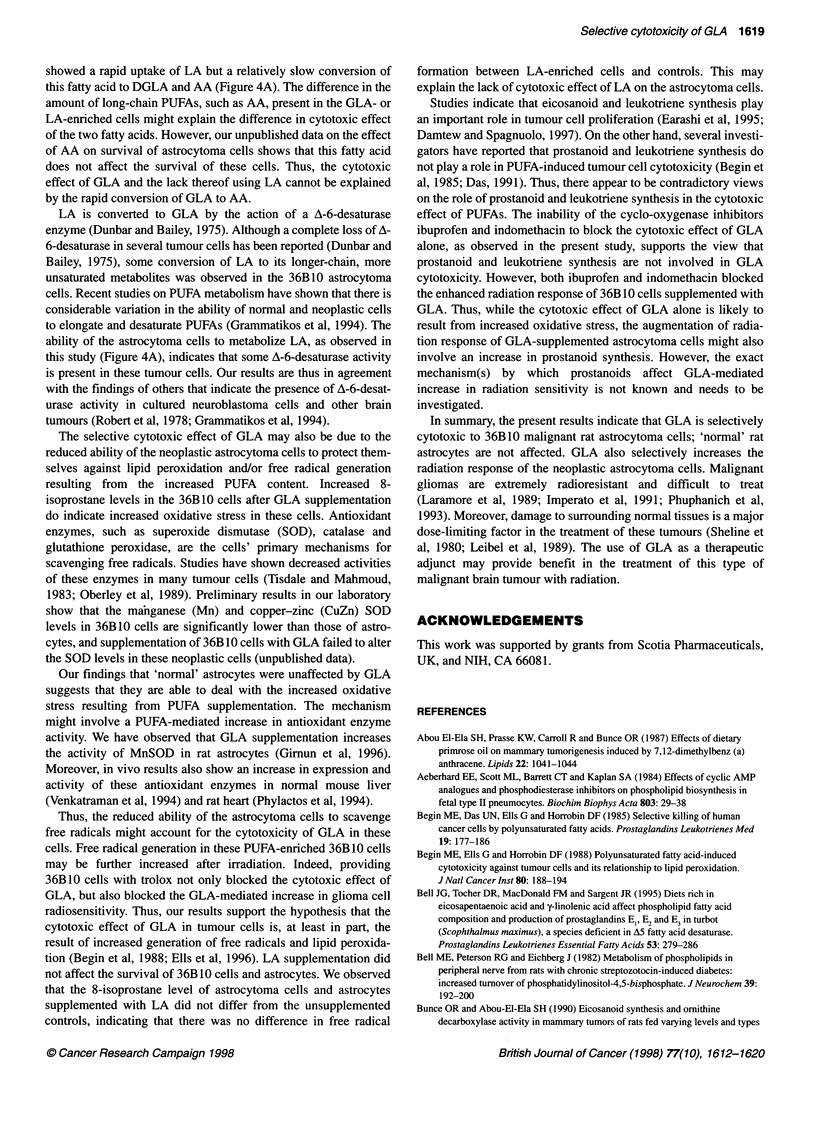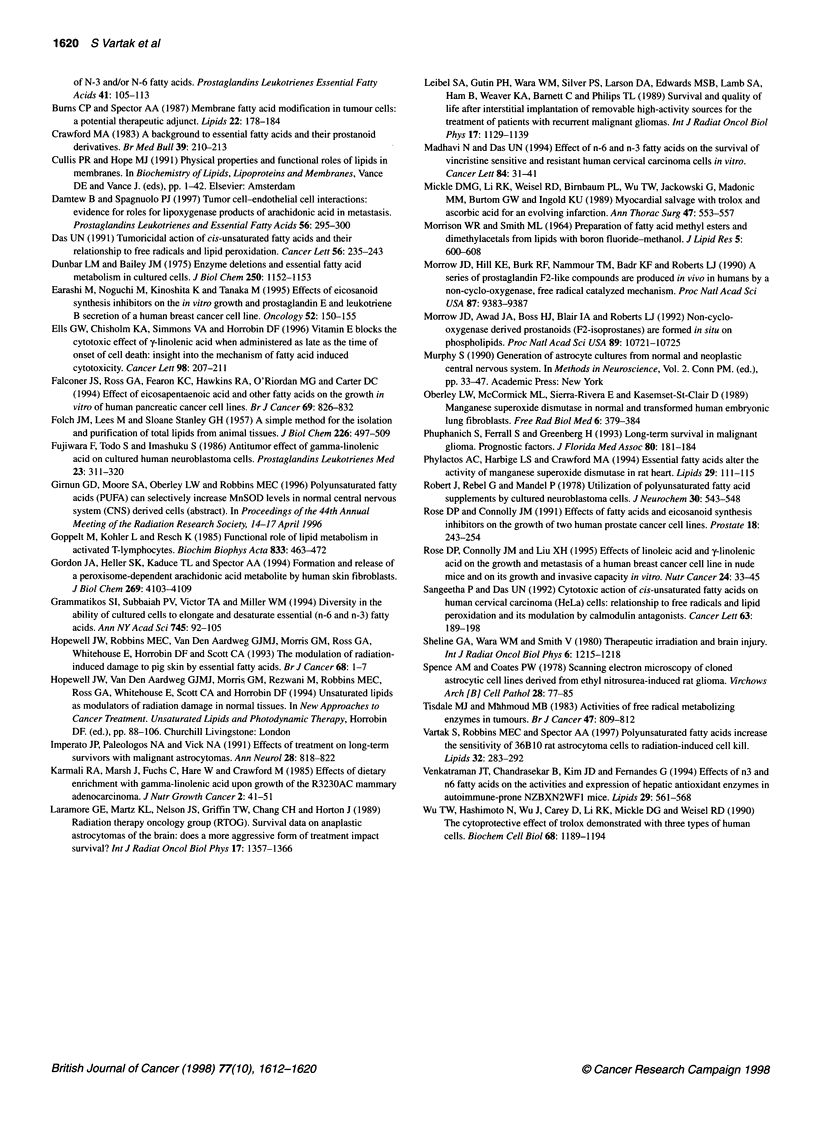# Gamma-linolenic acid (GLA) is cytotoxic to 36B10 malignant rat astrocytoma cells but not to 'normal' rat astrocytes.

**DOI:** 10.1038/bjc.1998.264

**Published:** 1998-05

**Authors:** S. Vartak, R. McCaw, C. S. Davis, M. E. Robbins, A. A. Spector

**Affiliations:** Radiation Research Laboratory, Department of Radiology, University of Iowa, Iowa City 52242, USA.

## Abstract

This study compares the effect of gamma-linolenic acid (GLA) and its precursor linoleic acid (LA) on survival of 36B10 malignant rat astrocytoma cells and 'normal' rat astrocytes. GLA was cytotoxic to 36B10 cells but not to astrocytes. By contrast, LA supplementation did not affect the survival of either cell types. There were minor differences in the uptake, distribution and use of radiolabelled GLA and LA by the 36B10 cells and astrocytes. GLA and LA supplementation increased the total polyunsaturated fatty acid (PUFA) content of the cells indicating increased oxidative potential. However, elevated levels of 8-isoprostane, an indicator of increased oxidative stress, were only observed in the GLA supplemented 36B10 cells. Addition of the antioxidant trolox to GLA-enriched 36B10 cells blocked the cytotoxic effect. Further, GLA enhanced the radiation sensitivity of the astrocytoma cells but not the astrocytes; trolox blocked the GLA-mediated increase in astrocytoma cell radiosensitivity. LA did not affect the radiation response of either cell type. While cyclo-oxygenase inhibitors did not affect GLA cytotoxicity, they blocked the enhanced radiation response of GLA-supplemented cells. The lipoxygenase inhibitor NDGA did not affect the toxicity produced by GLA. Thus, GLA is toxic to the neoplastic astrocytoma cells but not to normal astrocytes.


British Journal of Cancer (1998) 77(10), 1612–1620
© 1998 Cancer Research Campaign

### γ-Linolenic acid (GLA) is cytotoxic to 36B10 malignant rat astrocytoma cells but not to 'normal' rat astrocytes

**S Vartak[1], R McCaw[1], CS Davis[2], MEC Robbins[1] and AA Spector[3]**

[1]Radiation Research Laboratory, Department of Radiology, [2]Department of Preventive Medicine and [3]Department of Biochemistry, University of Iowa, Iowa City, IA 52242, USA

**Summary** This study compares the effect of γ-linolenic acid (GLA) and its precursor linoleic acid (LA) on survival of 36B10 malignant rat astrocytoma cells and 'normal' rat astrocytes. GLA was cytotoxic to 36B10 cells but not to astrocytes. By contrast, LA supplementation did not affect the survival of either cell types. There were minor differences in the uptake, distribution and use of radiolabelled GLA and LA by the 36B10 cells and astrocytes. GLA and LA supplementation increased the total polyunsaturated fatty acid (PUFA) content of the cells indicating increased oxidative potential. However, elevated levels of 8-isoprostane, an indicator of increased oxidative stress, were only observed in the GLA supplemented 36B10 cells. Addition of the antioxidant trolox to GLA-enriched 36B10 cells blocked the cytotoxic effect. Further, GLA enhanced the radiation sensitivity of the astrocytoma cells but not the astrocytes; trolox blocked the GLA-mediated increase in astrocytoma cell radiosensitivity. LA did not affect the radiation response of either cell type. While cyclo-oxygenase inhibitors did not affect GLA cytotoxicity, they blocked the enhanced radiation response of GLA-supplemented cells. The lipoxygenase inhibitor NDGA did not affect the toxicity produced by GLA. Thus, GLA is toxic to the neoplastic astrocytoma cells but not to normal astrocytes.

**Keywords:** polyunsaturated fatty acids; γ-linolenic acid; linoleic acid; astrocytoma; astrocyte; radiation

Gamma linolenic acid (GLA, 18:3n6) supplementation has been reported to suppress the growth of tumour cells in vitro (Fujiwara et al, 1986; Sangeetha and Das, 1992; Falconer et al, 1994) and in vivo (Karmali et al, 1985; Abou et al, 1987). Interestingly, in separate studies conducted on normal tissues in vivo, it has been reported that dietary GLA decreases the severity of radiation damage to the skin (Hopewell et al, 1993) and CNS (Hopewell et al, 1994). We have previously shown that GLA supplementation can decrease clonogenic cell survival of malignant rat astrocytoma cells and increase their radiosensitivity (Vartak et al, 1997). To determine the therapeutic potential of this observation, it is important to investigate the effect of GLA on 'normal' rat astrocytes. The purpose of this work was to compare the effects of GLA on the survival of 36B10 astrocytoma cells and astrocytes and their response to radiation. The uptake and use of GLA by astrocytoma cells has also been compared with that by astrocytes.

The cytotoxic action of polyunsaturated fatty acids (PUFAs) is thought to be mediated predominantly via lipid peroxidation and free radical generation (Begin et al, 1988; Ells et al, 1996). Several studies also indicate that eicosanoid and leukotriene synthesis play an important role in tumour cell proliferation and metastasis (Earashi et al, 1995; Damtew and Spagnuolo, 1997). Arachidonic acid (AA, 20:4n6) and dihomo-γ-linolenic acid (DGLA, 20:3n6) are the substrate for the biosynthesis of prostaglandins of the 2- and 1- series respectively (Crawford, 1983). GLA is metabolized to form DGLA and AA. Studies on the effect of PUFAs on eicosanoid synthesis have shown that GLA and other PUFAs increase prostanoid synthesis in cells (Bunce and Abou-El-Ela, 1990; Bell JG et al, 1995, Rose et al, 1995). In order to investigate the mechanism(s) by which GLA exerts a cytotoxic effect on astrocytoma cells, we have studied the role of lipid peroxidation and/or free radical generation, prostanoids and leukotrienes on the cytotoxic action of GLA and its enhancement of the response to radiation.

GLA is formed directly from linoleic acid (LA, 18:2n6) by the action of a delta 6-desaturase (Dunbar and Bailey, 1975). Therefore, we have also investigated whether LA would produce effects similar to those obtained with GLA.

*Received 13 August 1997*
*Revised 10 October 1997*
*Accepted 4 November 1997*

*Correspondence to:* S Vartak, Radiation Research Laboratory, 77C Medical Laboratories, University of Iowa, Iowa City, IA 52242, USA

### MATERIALS AND METHODS

### Cell and culture conditions

The tumour cell line tested was the ethylnitrosurea-induced 36B10 malignant rat astrocytoma (Spence and Coates, 1978). The 'normal' rat astrocytes used in the study were obtained as primary cell cultures isolated from 1- to 2-day-old Sprague–Dawley rat pups using the method of Murphy (1990). Briefly, cortices were removed from the rat pups, meninges were stripped and the cortices homogenized. After incubation with trypsin for 15 min in a 37°C shaking water bath, the homogenate was centrifuged at room temperature for 2 min at 1000 r.p.m. The supernatant was discarded and the pellet resuspended in a trypsin inhibitor/DNAase solution, triturated, layered over a bovine serum albumin (BSA) solution and centrifuged at room temperature for 7 min at 1000 r.p.m. The supernatant was removed and the pellet was resuspended in media. Cells were counted using a haemocytometer and plated at 56 000 cells $cm^{-2}$.

Both cell types were incubated at 37°C under a humidified atmosphere of 95% air:5% carbon dioxide. The astrocytoma cells were cultured in Dulbecco's modified Eagle medium containing

10% fetal bovine serum and L-glutamine (2 mM), penicillin (50 IU ml$^{-1}$) and streptomycin (50 µg ml$^{-1}$). They were maintained as monolayer cultures in tissue culture flasks by routine passage. The astrocytes were cultured in Eagle's minimum essential medium containing 10% fetal bovine serum and 2 mM L-glutamine, 33 mM glucose and 60 µg ml$^{-1}$ gentamycin. All cell culture reagents were purchased from Gibco, Grand Island, NY, USA.

### Cell survival analyses and cytotoxicity studies

The effect of GLA and LA, either alone or combined with radiation, on 36B10 astrocytoma cell survival (Cayman Chemical, Ann Arbor, MI, USA) was studied using the clonogenic cell survival assay. Viable cells assessed by the 0.4% erythrosin B dye exclusion method using a haemocytometer were seeded at varying densities depending on the radiation dose; 200 cells for unirradiated cells, 600 cells for 2.5 Gy, 1000 cells for 5 Gy, 1800 cells for 7.5 Gy and 2500 cells for 10 Gy ($^{137}$Cs γ-source; dose rate 1.5 Gy min$^{-1}$) in 60-mm dishes. At the end of a 7-day incubation period, they were stained with 0.1% crystal violet and the colonies (> 50 cells per colony) were counted using a dissecting microscope.

In order to study whether lipid peroxidation played a role in the effect of GLA on survival of 36B10 cells, 50 or 100 µM Trolox (Aldrich, Milwaukee, WI, USA) was added to the medium in addition to the GLA. Trolox is a water-soluble analogue of vitamin E and has been shown to protect mammalian cells from oxidative damage (Mickle et al, 1989; Wu et al, 1990). Stock solutions of trolox (100 mM) were prepared in 1 M sodium bicarbonate as, at concentrations above 1.8 mM, trolox has poor solubility in water. The pH was adjusted to 7.0 using 1 N hydrochloric acid.

To investigate whether altered prostaglandin or leukotriene synthesis was involved in GLA cytotoxicity and/or the modified radiation response of GLA-supplemented cells, the cyclo-oxygenase inhibitors ibuprofen (50–100 µM) or indomethacin (2.5–10 µM) and the lipoxygenase inhibitor nordihydroguaretic acid (NDGA, 1–5 µM) were used. All inhibitors were added to the medium at the same time as GLA, and the cells were maintained in this medium throughout the experiment. Ibuprofen, indomethacin and NDGA were purchased from Sigma Chemical, St Louis, MO, USA.

### Cell cytotoxicity studies for rat astrocytes

As rat astrocytes do not form colonies, the effect of fatty acids on the survival of these cells was determined using the fluorescent dye Calcein AM (Molecular Probes, Eugene, OR, USA). In order to facilitate direct comparison between the effects of GLA and LA on the survival of astrocytes and astrocytoma cells, this assay was also performed using the 36B10 cells.

Rat astrocytes (2500 cells per well) or astrocytoma cells (250 cells per well) were seeded onto 96-well plates. The two cell types were seeded at different densities because of the differences in their growth rates. The doubling time of rat astrocytes in culture is approximately 4 days, whereas that of the 36B10 astrocytoma cells is 16 h (unpublished data). After an overnight incubation, the cells were supplemented with 30 or 45 µM GLA or LA and/or exposed to 2.5–10 Gy γ-rays (1.5 Gy min$^{-1}$; $^{137}$Cs γ-source). The cells were then allowed to grow for 1–7 days at the end of which the viability of the cells was assessed. The cells were incubated with calcein-AM (live-cell fluorescence stain) at a final concentration of 1 µM for 45 min at room temperature. The fluorescence then was measured using a fluorescence microplate reader (FL500, Bio-Tek Instruments, VI, USA). The live cell populations were characterized by an intense fluorescence in the 530-nm region.

### Determination of 8-isoprostane levels

Astrocytoma cells and astrocytes were supplemented with 45 µM GLA or LA. The media was tested for 8-isoprostane 0–72 h after fatty acid supplementation. The isoprostanes are a family of eicosanoids of non-enzymatic origin produced through the random oxidation of tissue phospholipids by oxygen radicals. 8-Isoprostane (8-*epi*prostaglandin $F_{2\alpha}$) has been proposed as a marker of antioxidant deficiency and oxidative stress (Morrow et al, 1990, 1992). The level of 8-isoprostane after GLA and LA supplementation was measured using an EIA kit (Cayman Chemical). Briefly, the samples were incubated with the 8-isoprostane tracer (an 8-isoprostane-acetylcholinesterase conjugate) and 8-isoprostane polyclonal antiserum for 18 h at room temperature. After incubation, the plate was washed and developed for 60 min with Ellman's reagent (which contains the substrate for acetylcholinesterase). The plate was read at 405 nm using a microplate reader (CERESuv900c, Bio-Tek Instruments) and the amount of 8-isoprostane determined from an 8-isoprostane standard graph.

### Incorporation of GLA and LA into cells

Incorporation of GLA and LA into cells was determined after supplementation of the medium with 20 or 45 µM [1-$^{14}$C] 18:3 or [1-$^{14}$C] 18:2 for 1, 6, 24 and 48 h. Lipids were extracted from the cells using chloroform–methanol (2:1 v/v) following the standard protocol of Folch et al (1957). The radiolabelled fatty acids present in the media were determined by separating the media from the cells after the required incubations using 20 µM [1-$^{14}$C] GLA or LA. To determine the amount of radioactivity remaining in the medium and the amount incorporated into the cells, aliquots were taken from the cell extract and from the medium, after it had been centrifuged at 2500 r.p.m. for 5 min to remove any cell debris. Lipids were extracted from the medium using two volumes of water saturated ethyl acetate. The ethyl acetate was removed by evaporation under nitrogen and the fatty acids separated by high-performance liquid chromatography (HPLC). The lipid extracts were then transesterified with 12% boron fluoride in methanol at 95°C for 45 min (Morrison and Smith, 1964). The radiolabelled material was assayed using a liquid scintillation spectrophotometer (Packard Tricarb 4640). Quenching was monitored using an internal standard.

The phospholipid and neutral lipid fractions of the cell lipid extracts were separated by thin-layer chromatography (TLC) (Bell ME et al, 1982). Neutral lipids were separated on Whatman silica gel G plates with a mixture of heptane–diethyl ether–acetic acid–methanol (90:20:2:3). Phospholipids were separated on Whatman LK5D plates with a mixture of chloroform–methanol–40% methanolamine (60:36:5). The distribution of radioactivity on the TLC plate was determined with a gas flow proportional scanner (Radiomatic model R). Lipid standards or radiolabelled standards were applied to each plate and the chromatogram was visualized under UV light after development by spraying with 1 mM 8-anilino-1-naphthalene sulphonic acid, or analysed using the radioisotope scanner.

To measure the conversion of the fatty acids to metabolites, the cell and medium lipid extracts were analysed by reverse-phase

© Cancer Research Campaign 1998

**Figure 1** Time dependence of fatty acid uptake. Incorporation of 20 μM of [1-$^{14}$C] (**A**) GLA or (**B**) LA into 36B10 rat astrocytoma cells (○) and rat astrocytes (●). Cells were supplemented with the fatty acid for 1–48 h. Values represent the mean from two separate cultures

**Figure 2** Fatty acid incorporation into phospholipids. Distribution of 20 μM [1-$^{14}$C] GLA in the PC (○), PE (●), PI (△) and PS (▲) fractions of (**A**) 36B10 rat astrocytoma cells and (**B**) rat astrocytes. Cells were supplemented with the fatty acid for 1–48 h. Values represent the mean from two separate cultures

HPLC (Gordon et al, 1994). The HPLC was a Gilson 715 system comprising an 805 nanometric module, 811 dynamic mixer, 306 pump, 117 UV detector and a 231 XL sample injector. The column used was an Altech C18 2.1 × 150 mm Solvent Miser with 5-μm spherical packing. The elution profile for the separation of fatty acid methyl esters consisted of water adjusted to pH 3 with phosphoric acid and an acetonitrile gradient increasing from 60% to 100% over 65 min at a flow rate of 0.4 ml min$^{-1}$. Free fatty acid separation was achieved using an acetonitrile gradient that increased from 50% to 100% over 65 min at a flow rate of 0.4 ml min$^{-1}$. The radioactivity was measured by combining the column effluent with scintillator solution and passing the mixture through a Radiomatic Flow-one/Beta radioisotope detector A200 at 2 ml min$^{-1}$.

### Statistical analysis

Statistical analysis of the radiation response data was performed using simple linear regression. Simple linear regression models of the form 'log (surviving fraction) = β dose' were fitted. Student's *t*-tests were used to compare each slope to the control slope; *P*-values ≤ 0.05 were considered to be statistically significant. $D_0$ values, defined as the dose (Gy) required to reduce the fraction of surviving cells to 37% of its control value, were estimated by the negative reciprocal of the slope. Standard errors for $D_0$ values were calculated from the standard errors of the estimated slopes using Taylor series variance approximations (the delta method). Statistical significance for all other data was analysed using the Student's *t*-test. Data with *P*-values ≤ 0.05 were considered to be statistically significant.

### RESULTS

### Incorporation of GLA and LA into 36B10 cells and astrocytes

Figure 1 shows the incorporation of 20 μM [1-$^{14}$C] GLA (Figure 1A) and [1-$^{14}$C] LA (Figure 1B) into 36B10 astrocytoma cells and astrocytes. The incorporation of GLA and LA into both cell types was very rapid. The majority of the fatty acid available to the 36B10 cells was taken up in 6 h. However, the astrocytes continued to accumulate GLA and LA, and maximum incorporation did not occur until 24 h. Thus, at the end of the 48 h time period, the incorporation of the radiolabelled fatty acid by the astrocytes was ≥ 1.5 times greater than by the 36B10 cells. Similar

© Cancer Research Campaign 1998

**Figure 3** Conversion of 20 μM [1-$^{14}$C] GLA to other fatty acids by (**A**) 36B10 rat astrocytoma cells and (**B**) rat astrocytes. GLA (○), DGLA (●), AA (△) and DTA (▲). Cells were supplemented with GLA for 1–48 h. Values represent the mean from two separate cultures

**Figure 4** Conversion of 20 μM [1-$^{14}$C] LA to other fatty acids by (**A**) 36B10 rat astrocytoma cells and (**B**) rat astrocytes. LA (▲), GLA (○), DGLA (●) and AA (△). Cells were supplemented with LA for 1–48 h. Values represent the mean from two separate cultures results were obtained using 45 μM [1-$^{14}$C] GLA and LA (data not shown).

### Distribution of GLA and LA in 36B10 astrocytoma cells and astrocytes

Using TLC it was determined that the majority of the total radiolabelled fatty acids taken up by the 36B10 cells and astrocytes supplemented with either fatty acids was present in the phospholipids. Figure 2 shows the distribution of [1-$^{14}$C] GLA into the different phospholipid classes of the astrocytoma cells (Figure 2A) and astrocytes (Figure 2B) between 1 and 48 h after GLA supplementation. The two cell types showed some differences in the distribution of the fatty acid in the various phospholipid classes. In the 36B10 cells, phosphatidylcholine (PC) contained approximately 60% of the radioactivity during the first 6 h. Subsequently, the amount of radioactivity in PC declined and phosphatidylethanolamine (PE) became the most heavily labelled fraction (Figure 2A). Thus, at 48 h, PE contained 60% of the radioactivity, with only 19% remaining in the PC. The amounts of phosphatidylinositol (PI) and phosphatidylserine (PS) also showed small increases with time. In the case of the astrocytes, however, PC contained most of the radioactivity at all the time points studied (Figure 2B). The amount of radioactivity in the remaining phospholipid classes showed small increases with time.

Similar patterns of distribution were noticed when 36B10 cells and astrocytes were supplemented with LA (data not shown).

### Metabolism of GLA and LA by 36B10 cells and astrocytes

Figure 3 shows the conversion of [1–$^{14}$C] GLA to other radiolabelled fatty acids by the 36B10 cells and astrocytes. The 36B10 cells rapidly converted GLA to DGLA and AA; by 24 h, all of the [1-$^{14}$C] GLA supplied was metabolized mainly to AA (Figure 3A). On the other hand, the astrocytes quickly converted the GLA to DGLA, AA and docosatetraenoic acid (DTA, 22:4n6; Figure 3B). As in the case of the 36B10 cells, AA was the predominant product in the astrocytes from 6 to 48 h GLA supplementation.

For comparision, the metabolism of [1-$^{14}$C] LA by the astrocytoma cells and astrocytes is shown in Figure 4. Although the pattern of use of LA by the 36B10 cells and astrocytes was similar, the rate of conversion of LA to its metabolites was different for the two cell types. Most of the LA supplied to the astrocytoma cells remained as LA, with little conversion to DGLA and AA (Figure

**Figure 5** Effect of fatty acid incorporation on astrocytoma cell survival and response to radiation. **A** shows the effect of GLA and LA on the survival of 36B10 cells. Cells were supplemented with 30 µM (▨) or 45 µM (■) GLA or LA throughout the duration of the experiment (7 days). Control cultures (□) received no supplement. Values represent mean ± s.e. obtained from two experiments ($n = 6$). **B** shows the effect of GLA and LA on the radiation response of 36B10 cells. Cells supplemented with 45 µM GLA (●) or 45 µM LA (△) for 24 h were irradiated with 0- to 10-Gy γ-radiation. Cells were continued on supplemented media throughout the experiment. Control cultures (○) received no supplement. Values represent mean ± s.e. obtained from two experiments ($n = 6$). **Significantly different from controls; $P < 0.01$

**Figure 6** Comparison of the effects of GLA on the survival and radiation responses of astrocytoma cells and astrocytes. Effect of 30 or 45 µM GLA supplementation on (**A**) survival and (**B**) radiation response at 7.5 Gy of 36B10 cells (□) and astrocytes (■) was assessed using the calcein AM fluorescence assay. Cells were supplemented with the fatty acid for 7 days. Bars indicate values obtained from one experiment with $n = 5$ (mean ± s.e.). *$P < 0.05$ and **$P < 0.01$; significantly different from respective controls

4A). The astrocytes, however, readily converted LA to its longer chain metabolites (Figure 4B).

### Effect of GLA and LA on the survival and radiation response of 36B10 cells and rat astrocytes

Figure 5A and B show the effect of LA or GLA supplementation on the survival and the radiation response of 36B10 cells as assessed using the clonogenic cell survival assay. Incubating the cells with 45 µM GLA reduced their survival to 55% ($P < 0.01$) of the controls, which received no fatty acid supplement (Figure 5A). GLA supplementation (45 µM) also significantly enhanced the radiation response of the 36B10 cells ($P < 0.01$; Figure 5B). As previously published (Vartak et al, 1997), the mean (± s.e.) $D_0$ Gy value obtained for cells supplemented with GLA was significantly reduced ($P < 0.001$; $D_0$ Gy = 1.92 ± 0.04) compared with that of the cells that received no fatty acid supplement ($D_0$ Gy = 2.70 ± 0.04). In marked contrast, LA supplementation did not affect either survival of the astrocytoma cells or the response of the astrocytoma cells to radiation. In fact, the $D_0$ value obtained for cells enriched with LA ($D_0$ Gy = 3.00 ± 0.14) was higher than that for the unsupplemented controls.

Figure 6 shows the effect of GLA (Figure 6A) and GLA plus 7.5-Gy radiation (Figure 6B) on the survival of 'normal' rat astrocytes compared with the rat astrocytoma cells, assessed using the fluorescent dye calcein AM. Consistent with the clonogenic assay, supplementation of 36B10 cells with 45 µM GLA alone reduced their survival to 65 ± 1.59% of the controls ($P < 0.01$; Figure 6A). However, GLA had no effect on the viability of the astrocytes. Similarly, GLA at 45 µM enhanced the radiation response of the astrocytoma cells but not that of the astrocytes (Figure 6A and B). In contrast, supplementation of the neoplastic astrocytoma cells or astrocytes with LA did not affect their survival or radiation response as assessed by the fluorescent dye assay (data not shown).

### Effect of trolox and GLA on survival and radiation response of astrocytoma cells

Figure 7A shows the effects of trolox on the survival of 36B10 cells supplemented with GLA. Incubation of the cells with 50 or 100 µM trolox alone did not affect their survival; as previously noted (Vartak et al, 1997), GLA (45 µM) supplementation reduced survival to 55 ± 8.8% of control values ($P < 0.01$). However, when the cells were supplemented with trolox in addition to the GLA, the cytotoxic effect of GLA was completely blocked. Similarly,

*© Cancer Research Campaign 1998*

**Figure 7** Trolox protects the GLA-mediated increase in 36B10 cell cytotoxicity and radiation response. **A** shows the effect of GLA and trolox on the survival of 36B10 cells. Cells were supplemented with 50 μM (▨) or 100 μM (▩) trolox alone, 45 μM GLA alone (▤), GLA and 50 μM trolox (▦) or GLA and 100 μM trolox (■) throughout the duration of the experiment (7 days). Control cultures (□) received no supplement. Values represent mean ± s.e. obtained from two experiments ($n = 6$). **B** shows the effect of GLA and trolox on the radiation response of 36B10 cells. Cells supplemented with 50 (●) or 100 (△) μM trolox alone, 45 μM GLA alone (▲), GLA and 50 μM trolox (□) or GLA and 100 μM trolox (■) for 24 h were irradiated with 0- to 10-Gy γ-radiation. Cells were continued on supplemented media throughout the experiment. Control cultures (○) received no supplement. Values represent mean ± s.e. obtained from two experiments ($n = 6$). Cells supplemented with 50 (●) or 100 (△) μM ibuprofen alone, 45 μM GLA alone (▲), GLA and 50 μM ibuprofen (□) or GLA and 100 μM ibuprofen (■) for 24 h were irradiated with 0- to 10-Gy γ-radiation. **Significantly different from controls; $P < 0.01$

**Figure 8** 8-Isoprostane production in (**A**) 36B10 cells and (**B**) astrocytes supplemented with GLA or LA. Cells were supplemented with 45 μM GLA (●) or LA (△) and the 8-isoprostane levels were measured 0–72 h after supplementation. Control cells (○) received no GLA supplement. Values represent mean ± s.e. from one experiment with $n = 4$. *$P < 0.05$ and **$P < 0.01$; significantly different from respective controls addition of trolox along with GLA led to a complete inhibition of the enhancement of radiation response observed in GLA-supplemented cells (Figure 7B).

### 8-Isoprostane levels of 36B10 cells supplemented with GLA

As the protective effect of trolox suggested that GLA might act through the production of oxidative products, we determined whether 8-isoprostane production increased in the supplemented cells. Figure 8 shows the effect of 45 μM GLA and LA supplementation on the 8-isoprostane level in 36B10 cells and astrocytes 24–72 h after incubation with the fatty acids. Supplementation of the astrocytoma cells with GLA for 72 h increased the level of 8-isoprostane approximately threefold ($P < 0.01$), but LA produced no increase compared with control unsupplemented cells (Figure 8A). However, the astrocytes exhibited a smaller increase in 8-isoprostane level after incubation with GLA compared with the increase observed in 36B10 cells (compare Figure 8A and B). Further, LA supplementation did not alter the 8-isoprostane level of the astrocytes.

### Effect of cyclo-oxygenase and lipoxygenase inhibitors on the radiation response of GLA-supplemented 36B10 cells

In order to investigate whether prostanoid and/or leukotriene synthesis is involved in the cytotoxic effect of GLA and its enhancement of the radiation response in the neoplastic cells, the cyclo-oxygenase inhibitors ibuprofen and indomethacin and the lipoxygenase inhibitor NDGA were tested. Ibuprofen alone had no effect on the survival of the astrocytoma cells and addition of ibuprofen plus GLA did not affect GLA cytotoxicity (Figure 9A). However, incubating the cells with ibuprofen along with GLA completely blocked the GLA-induced enhancement of radiation response of 36B10 cells (Figure 9B). Similar results were obtained with 2.5–10 μM indomethacin (data not shown).

© Cancer Research Campaign 1998

**Figure 9** Cyclo-oxygenase inhibitor prevents the GLA enhancement of the radiation response but not GLA-mediated cytotoxicity. **A** shows the effect of 45 μM GLA and 50–100 μM ibuprofen on the survival of 36B10 cells. Cells were supplemented with 50 μM (▨) or 100 μM (⊠) ibuprofen alone, 45 μM GLA alone (⊟), GLA and 50 μM ibuprofen (⊞) or GLA and 100 μM ibuprofen (■) throughout the duration of the experiment (7 days). Control cultures (□) received no supplement. Values represent mean ± s.e. obtained from two experiments ($n$ = 6). **B** shows the effect of GLA and ibuprofen on the radiation response of 36B10 cells. Cells supplemented with 50 (●) or 100 μM (△) ibuprofen alone, 45 μM GLA alone (▲), GLA and 50 μM ibuprofen (□) or GLA and 100 μM ibuprofen (■) for 24 h were irradiated with 0- to 10-Gy γ-radiation. Cells were continued on supplemented media throughout the experiment. Control cultures (○) received no supplement. Values represent mean ± s.e. obtained from two experiments ($n$ = 6). **Significantly different from controls; $P < 0.01$

The lipoxygenase inhibitor NDGA, at concentrations of 1–5 μM, did not affect the survival of the astrocytoma cells (data not shown). Further, incubation of the astrocytoma cells with GLA and NDGA did not affect GLA cytotoxicity or the GLA-induced increase in radiation sensitivity (data not shown).

### DISCUSSION

The present results show that the overall pattern of distribution and metabolism of GLA and LA by 36B10 malignant rat astrocytoma cells differs little from that of the astrocytes. However, GLA is cytotoxic to the astrocytoma cells but not to the 'normal' astrocytes. In contrast, the precursor of GLA, LA, is not cytotoxic to either 36B10 cells or astrocytes. The increase in 8-isoprostane formation after GLA supplementation and the protective effect of trolox in the astrocytoma cells suggest that the cytotoxic action of GLA alone and the GLA-mediated increase in radiation-induced cytotoxicity is the result of increased lipid peroxidation and/or free radical generation. However, the ability of the cyclo-oxygenase inhibitors ibuprofen and indomethacin to block the GLA-mediated increase in astrocytoma cell radiosensitivity suggests that, in addition to the increased oxidative stress, this effect may involve increased prostanoid synthesis.

Changes in fatty acid composition as a result of alterations in the type of fatty acid available have been reported in vivo and in vitro (Burns and Spector, 1987). Indeed, we have previously demonstrated marked changes in the fatty acid profiles of 36B10 cells supplemented with GLA (Vartak et al, 1997). We have tested the ability of the cells to use this fatty acid in the present study. Our results show that both neoplastic astrocytoma cells and 'normal' astrocytes are able to incorporate GLA and LA. The amount of GLA or LA incorporated into the astrocytes was greater than the 36B10 cells. However, we have observed that GLA was toxic to the 36B10 cells; astrocytes were unaffected. Thus, the selectively cytotoxic effect of GLA cannot be explained based on differences in uptake of GLA by the cells.

A difference in the distribution of the fatty acids into the various phospholipid classes was also observed for the two cell types. All GLA or LA supplied to the astrocytoma cells was first incorporated into the PC fraction, with a gradual shift to PE over time (Figure 2A). However, this shift was much more dramatic in the GLA-supplemented astrocytoma cells than when they were incubated in LA-enriched medium. Studies conducted on the distribution of the different phospholipid classes in various mammalian cell membranes show that the outer leaflet of the lipid bilayer predominantly consists of PC while the majority of the PE is confined to the inner leaflet (Cullis and Hope, 1991). Thus, the shift of radiolabelled fatty acid from PC to PE observed in our study may simply reflect a redistribution of the newly incorporated fatty acids from the outer to the inner leaflet of the cell membrane. The shift of radioactivity from the PC to the PE fraction may also be due to increased amounts of AA in the cells. Unsaturated fatty acids are incorporated preferentially with regard to individual phospholipid classes. While OA and LA are found primarily in the PC fraction, AA is transferred to both PE and PI. Several groups have reported a redistribution of AA from PC to PE when AA was provided to cells (Aeberhard et al, 1984; Goppelt et al, 1985). We observed that the 36B10 cells rapidly converted the GLA supplied to AA; the transfer of radioactivity from the PC to the PE fraction may reflect this conversion of GLA to AA. In addition, the relatively slower shift of radioactivity from PC to PE in LA-supplemented cells observed in the present study might be because of the slower conversion of LA to AA by the astrocytoma. The astrocytes, however, incorporated most of the fatty acid supplied into the PC fraction with little shift to PE over the time course studied (Figure 2B). While the exact reason for this remains unclear, the results do suggest that the two cell types incorporate the fatty acid supplied into different membrane compartments.

Our results show that GLA is selectively toxic to the tumour cells, whereas it has no deleterious effect on astrocyte survival. In contrast, LA has no toxic effect on either of these cell populations. Studies on several different neoplastic cell types also indicate that GLA has a greater cytotoxic effect than LA (Fujiwara et al, 1986; Begin et al, 1998; Connolly, 1991; Madhavi and Das, 1994). Our results suggest that the difference in the cytotoxicity of GLA and LA might be due, in part, to differences in their metabolism by the astrocytoma cells. While the 36B10 cells could rapidly elongate and desaturate GLA to DGLA and AA (Figure 3A), these cells

 *© Cancer Research Campaign 1998*

showed a rapid uptake of LA but a relatively slow conversion of this fatty acid to DGLA and AA (Figure 4A). The difference in the amount of long-chain PUFAs, such as AA, present in the GLA- or LA-enriched cells might explain the difference in cytotoxic effect of the two fatty acids. However, our unpublished data on the effect of AA on survival of astrocytoma cells shows that this fatty acid does not affect the survival of these cells. Thus, the cytotoxic effect of GLA and the lack thereof using LA cannot be explained by the rapid conversion of GLA to AA.

LA is converted to GLA by the action of a Δ-6-desaturase enzyme (Dunbar and Bailey, 1975). Although a complete loss of Δ-6-desaturase in several tumour cells has been reported (Dunbar and Bailey, 1975), some conversion of LA to its longer-chain, more unsaturated metabolites was observed in the 36B10 astrocytoma cells. Recent studies on PUFA metabolism have shown that there is considerable variation in the ability of normal and neoplastic cells to elongate and desaturate PUFAs (Grammatikos et al, 1994). The ability of the astrocytoma cells to metabolize LA, as observed in this study (Figure 4A), indicates that some Δ-6-desaturase activity is present in these tumour cells. Our results are thus in agreement with the findings of others that indicate the presence of Δ-6-desaturase activity in cultured neuroblastoma cells and other brain tumours (Robert et al, 1978; Grammatikos et al, 1994).

The selective cytotoxic effect of GLA may also be due to the reduced ability of the neoplastic astrocytoma cells to protect themselves against lipid peroxidation and/or free radical generation resulting from the increased PUFA content. Increased 8-isoprostane levels in the 36B10 cells after GLA supplementation do indicate increased oxidative stress in these cells. Antioxidant enzymes, such as superoxide dismutase (SOD), catalase and glutathione peroxidase, are the cells' primary mechanisms for scavenging free radicals. Studies have shown decreased activities of these enzymes in many tumour cells (Tisdale and Mahmoud, 1983; Oberley et al, 1989). Preliminary results in our laboratory show that the manganese (Mn) and copper–zinc (CuZn) SOD levels in 36B10 cells are significantly lower than those of astrocytes, and supplementation of 36B10 cells with GLA failed to alter the SOD levels in these neoplastic cells (unpublished data).

Our findings that 'normal' astrocytes were unaffected by GLA suggests that they are able to deal with the increased oxidative stress resulting from PUFA supplementation. The mechanism might involve a PUFA-mediated increase in antioxidant enzyme activity. We have observed that GLA supplementation increases the activity of MnSOD in rat astrocytes (Girnun et al, 1996). Moreover, in vivo results also show an increase in expression and activity of these antioxidant enzymes in normal mouse liver (Venkatraman et al, 1994) and rat heart (Phylactos et al, 1994).

Thus, the reduced ability of the astrocytoma cells to scavenge free radicals might account for the cytotoxicity of GLA in these cells. Free radical generation in these PUFA-enriched 36B10 cells may be further increased after irradiation. Indeed, providing 36B10 cells with trolox not only blocked the cytotoxic effect of GLA, but also blocked the GLA-mediated increase in glioma cell radiosensitivity. Thus, our results support the hypothesis that the cytotoxic effect of GLA in tumour cells is, at least in part, the result of increased generation of free radicals and lipid peroxidation (Begin et al, 1988; Ells et al, 1996). LA supplementation did not affect the survival of 36B10 cells and astrocytes. We observed that the 8-isoprostane level of astrocytoma cells and astrocytes supplemented with LA did not differ from the unsupplemented controls, indicating that there was no difference in free radical formation between LA-enriched cells and controls. This may explain the lack of cytotoxic effect of LA on the astrocytoma cells.

Studies indicate that eicosanoid and leukotriene synthesis play an important role in tumour cell proliferation (Earashi et al, 1995; Damtew and Spagnuolo, 1997). On the other hand, several investigators have reported that prostanoid and leukotriene synthesis do not play a role in PUFA-induced tumour cell cytotoxicity (Begin et al, 1985; Das, 1991). Thus, there appear to be contradictory views on the role of prostanoid and leukotriene synthesis in the cytotoxic effect of PUFAs. The inability of the cyclo-oxygenase inhibitors ibuprofen and indomethacin to block the cytotoxic effect of GLA alone, as observed in the present study, supports the view that prostanoid and leukotriene synthesis are not involved in GLA cytotoxicity. However, both ibuprofen and indomethacin blocked the enhanced radiation response of 36B10 cells supplemented with GLA. Thus, while the cytotoxic effect of GLA alone is likely to result from increased oxidative stress, the augmentation of radiation response of GLA-supplemented astrocytoma cells might also involve an increase in prostanoid synthesis. However, the exact mechanism(s) by which prostanoids affect GLA-mediated increase in radiation sensitivity is not known and needs to be investigated.

In summary, the present results indicate that GLA is selectively cytotoxic to 36B10 malignant rat astrocytoma cells; 'normal' rat astrocytes are not affected. GLA also selectively increases the radiation response of the neoplastic astrocytoma cells. Malignant gliomas are extremely radioresistant and difficult to treat (Laramore et al, 1989; Imperato et al, 1991; Phuphanich et al, 1993). Moreover, damage to surrounding normal tissues is a major dose-limiting factor in the treatment of these tumours (Sheline et al, 1980; Leibel et al, 1989). The use of GLA as a therapeutic adjunct may provide benefit in the treatment of this type of malignant brain tumour with radiation.

### ACKNOWLEDGEMENTS

This work was supported by grants from Scotia Pharmaceuticals, UK, and NIH, CA 66081.

### REFERENCES

Abou El-Ela SH, Prasse KW, Carroll R and Bunce OR (1987) Effects of dietary primrose oil on mammary tumorigenesis induced by 7,12-dimethylbenz (a) anthracene. *Lipids* **22**: 1041–1044

Aeberhard EE, Scott ML, Barrett CT and Kaplan SA (1984) Effects of cyclic AMP analogues and phosphodiesterase inhibitors on phospholipid biosynthesis in fetal type II pneumocytes. *Biochim Biophys Acta* **803**: 29–38

Begin ME, Das UN, Ells G and Horrobin DF (1985) Selective killing of human cancer cells by polyunsaturated fatty acids. *Prostaglandins Leukotrienes Med* **19**: 177–186

Begin ME, Ells G and Horrobin DF (1988) Polyunsaturated fatty acid-induced cytotoxicity against tumour cells and its relationship to lipid peroxidation. *J Natl Cancer Inst* **80**: 188–194

Bell JG, Tocher DR, MacDonald FM and Sargent JR (1995) Diets rich in eicosapentaenoic acid and γ-linolenic acid affect phospholipid fatty acid composition and production of prostaglandins $E_1$, $E_2$ and $E_3$ in turbot (*Scophthalmus maximus*), a species deficient in Δ5 fatty acid desaturase. *Prostaglandins Leukotrienes Essential Fatty Acids* **53**: 279–286

Bell ME, Peterson RG and Eichberg J (1982) Metabolism of phospholipids in peripheral nerve from rats with chronic streptozotocin-induced diabetes: increased turnover of phosphatidylinositol-4,5-*bis*phosphate. *J Neurochem* **39**: 192–200

Bunce OR and Abou-El-Ela SH (1990) Eicosanoid synthesis and ornithine decarboxylase activity in mammary tumors of rats fed varying levels and types

© Cancer Research Campaign 1998

of N-3 and/or N-6 fatty acids. *Prostaglandins Leukotrienes Essential Fatty Acids* **41**: 105–113

Burns CP and Spector AA (1987) Membrane fatty acid modification in tumour cells: a potential therapeutic adjunct. *Lipids* **22**: 178–184

Crawford MA (1983) A background to essential fatty acids and their prostanoid derivatives. *Br Med Bull* **39**: 210–213

Cullis PR and Hope MJ (1991) Physical properties and functional roles of lipids in membranes. In *Biochemistry of Lipids, Lipoproteins and Membranes*, Vance DE and Vance J. (eds), pp. 1–42. Elsevier: Amsterdam

Damtew B and Spagnuolo PJ (1997) Tumor cell–endothelial cell interactions: evidence for roles for lipoxygenase products of arachidonic acid in metastasis. *Prostaglandins Leukotrienes and Essential Fatty Acids* **56**: 295–300

Das UN (1991) Tumoricidal action of *cis*-unsaturated fatty acids and their relationship to free radicals and lipid peroxidation. *Cancer Lett* **56**: 235–243

Dunbar LM and Bailey JM (1975) Enzyme deletions and essential fatty acid metabolism in cultured cells. *J Biol Chem* **250**: 1152–1153

Earashi M, Noguchi M, Kinoshita K and Tanaka M (1995) Effects of eicosanoid synthesis inhibitors on the *in vitro* growth and prostaglandin E and leukotriene B secretion of a human breast cancer cell line. *Oncology* **52**: 150–155

Ells GW, Chisholm KA, Simmons VA and Horrobin DF (1996) Vitamin E blocks the cytotoxic effect of γ-linolenic acid when administered as late as the time of onset of cell death: insight into the mechanism of fatty acid induced cytotoxicity. *Cancer Lett* **98**: 207–211

Falconer JS, Ross GA, Fearon KC, Hawkins RA, O'Riordan MG and Carter DC (1994) Effect of eicosapentaenoic acid and other fatty acids on the growth *in vitro* of human pancreatic cancer cell lines. *Br J Cancer* **69**: 826–832

Folch JM, Lees M and Sloane Stanley GH (1957) A simple method for the isolation and purification of total lipids from animal tissues. *J Biol Chem* **226**: 497–509

Fujiwara F, Todo S and Imashuku S (1986) Antitumor effect of gamma-linolenic acid on cultured human neuroblastoma cells. *Prostaglandins Leukotrienes Med* **23**: 311–320

Girnun GD, Moore SA, Oberley LW and Robbins MEC (1996) Polyunsaturated fatty acids (PUFA) can selectively increase MnSOD levels in normal central nervous system (CNS) derived cells (abstract). In *Proceedings of the 44th Annual Meeting of the Radiation Research Society, 14–17 April 1996*

Goppelt M, Kohler L and Resch K (1985) Functional role of lipid metabolism in activated T-lymphocytes. *Biochim Biophys Acta* **833**: 463–472

Gordon JA, Heller SK, Kaduce TL and Spector AA (1994) Formation and release of a peroxisome-dependent arachidonic acid metabolite by human skin fibroblasts. *J Biol Chem* **269**: 4103–4109

Grammatikos SI, Subbaiah PV, Victor TA and Miller WM (1994) Diversity in the ability of cultured cells to elongate and desaturate essential (n-6 and n-3) fatty acids. *Ann NY Acad Sci* **745**: 92–105

Hopewell JW, Robbins MEC, Van Den Aardweg GJMJ, Morris GM, Ross GA, Whitehouse E, Horrobin DF and Scott CA (1993) The modulation of radiation-induced damage to pig skin by essential fatty acids. *Br J Cancer* **68**: 1–7

Hopewell JW, Van Den Aardweg GJMJ, Morris GM, Rezwani M, Robbins MEC, Ross GA, Whitehouse E, Scott CA and Horrobin DF (1994) Unsaturated lipids as modulators of radiation damage in normal tissues. In *New Approaches to Cancer Treatment. Unsaturated Lipids and Photodynamic Therapy*, Horrobin DF. (ed.), pp. 88–106. Churchill Livingstone: London

Imperato JP, Paleologos NA and Vick NA (1991) Effects of treatment on long-term survivors with malignant astrocytomas. *Ann Neurol* **28**: 818–822

Karmali RA, Marsh J, Fuchs C, Hare W and Crawford M (1985) Effects of dietary enrichment with gamma-linolenic acid upon growth of the R3230AC mammary adenocarcinoma. *J Nutr Growth Cancer* **2**: 41–51

Laramore GE, Martz KL, Nelson JS, Griffin TW, Chang CH and Horton J (1989) Radiation therapy oncology group (RTOG). Survival data on anaplastic astrocytomas of the brain: does a more aggressive form of treatment impact survival? *Int J Radiat Oncol Biol Phys* **17**: 1357–1366

Leibel SA, Gutin PH, Wara WM, Silver PS, Larson DA, Edwards MSB, Lamb SA, Ham B, Weaver KA, Barnett C and Philips TL (1989) Survival and quality of life after interstitial implantation of removable high-activity sources for the treatment of patients with recurrent malignant gliomas. *Int J Radiat Oncol Biol Phys* **17**: 1129–1139

Madhavi N and Das UN (1994) Effect of n-6 and n-3 fatty acids on the survival of vincristine sensitive and resistant human cervical carcinoma cells *in vitro*. *Cancer Lett* **84**: 31–41

Mickle DMG, Li RK, Weisel RD, Birnbaum PL, Wu TW, Jackowski G, Madonic MM, Burtom GW and Ingold KU (1989) Myocardial salvage with trolox and ascorbic acid for an evolving infarction. *Ann Thorac Surg* **47**: 553–557

Morrison WR and Smith ML (1964) Preparation of fatty acid methyl esters and dimethylacetals from lipids with boron fluoride–methanol. *J Lipid Res* **5**: 600–608

Morrow JD, Hill KE, Burk RF, Nammour TM, Badr KF and Roberts LJ (1990) A series of prostaglandin F2-like compounds are produced *in vivo* in humans by a non-cyclo-oxygenase, free radical catalyzed mechanism. *Proc Natl Acad Sci USA* **87**: 9383–9387

Morrow JD, Awad JA, Boss HJ, Blair IA and Roberts LJ (1992) Non-cyclo-oxygenase derived prostanoids (F2-isoprostanes) are formed *in situ* on phospholipids. *Proc Natl Acad Sci USA* **89**: 10721–10725

Murphy S (1990) Generation of astrocyte cultures from normal and neoplastic central nervous system. In *Methods in Neuroscience*, Vol. 2. Conn PM. (ed.), pp. 33–47. Academic Press: New York

Oberley LW, McCormick ML, Sierra-Rivera E and Kasemset-St-Clair D (1989) Manganese superoxide dismutase in normal and transformed human embryonic lung fibroblasts. *Free Rad Biol Med* **6**: 379–384

Phuphanich S, Ferrall S and Greenberg H (1993) Long-term survival in malignant glioma. Prognostic factors. *J Florida Med Assoc* **80**: 181–184

Phylactos AC, Harbige LS and Crawford MA (1994) Essential fatty acids alter the activity of manganese superoxide dismutase in rat heart. *Lipids* **29**: 111–115

Robert J, Rebel G and Mandel P (1978) Utilization of polyunsaturated fatty acid supplements by cultured neuroblastoma cells. *J Neurochem* **30**: 543–548

Rose DP and Connolly JM (1991) Effects of fatty acids and eicosanoid synthesis inhibitors on the growth of two human prostate cancer cell lines. *Prostate* **18**: 243–254

Rose DP, Connolly JM and Liu XH (1995) Effects of linoleic acid and γ-linolenic acid on the growth and metastasis of a human breast cancer cell line in nude mice and on its growth and invasive capacity *in vitro*. *Nutr Cancer* **24**: 33–45

Sangeetha P and Das UN (1992) Cytotoxic action of *cis*-unsaturated fatty acids on human cervical carcinoma (HeLa) cells: relationship to free radicals and lipid peroxidation and its modulation by calmodulin antagonists. *Cancer Lett* **63**: 189–198

Sheline GA, Wara WM and Smith V (1980) Therapeutic irradiation and brain injury. *Int J Radiat Oncol Biol Phys* **6**: 1215–1218

Spence AM and Coates PW (1978) Scanning electron microscopy of cloned astrocytic cell lines derived from ethyl nitrosurea-induced rat glioma. *Virchows Arch [B] Cell Pathol* **28**: 77–85

Tisdale MJ and Mahmoud MB (1983) Activities of free radical metabolizing enzymes in tumours. *Br J Cancer* **47**: 809–812

Vartak S, Robbins MEC and Spector AA (1997) Polyunsaturated fatty acids increase the sensitivity of 36B10 rat astrocytoma cells to radiation-induced cell kill. *Lipids* **32**: 283–292

Venkatraman JT, Chandrasekar B, Kim JD and Fernandes G (1994) Effects of n3 and n6 fatty acids on the activities and expression of hepatic antioxidant enzymes in autoimmune-prone NZBXN2WF1 mice. *Lipids* **29**: 561–568

Wu TW, Hashimoto N, Wu J, Carey D, Li RK, Mickle DG and Weisel RD (1990) The cytoprotective effect of trolox demonstrated with three types of human cells. *Biochem Cell Biol* **68**: 1189–1194

© Cancer Research Campaign 1998